# Supplementary material for: Quantum emission from coupled spin pairs in hexagonal boron nitride
Source: Nat Commun. 2025 Jul 1;16:5842. doi: 10.1038/s41467-025-61388-8 (PMC12217494; doi:10.1038/s41467-025-61388-8)
Supplement: Supplementary file 1 — Supplementary Information [file 41467_2025_61388_MOESM1_ESM.pdf]

Supplementary Materials for Quantum Emission from Coupled Spin Pairs in Hexagonal Boron Nitride

Song Li,[1,2,*] Anton Pershin,[1,3] and Adam Gali[1,3,4,†]

[1] *Wigner Research Centre for Physics, P.O. Box 49, H-1525 Budapest, Hungary*
[2] *Beijing Computational Science Research Center, Beijing 100193, China*
[3] *Department of Atomic Physics, Institute of Physics, Budapest University of Technology and Economics, Műegyetem rakpart 3., H-1111 Budapest, Hungary*
[4] *MTA–WFK Lendület "Momentum" Semiconductor Nanostructures Research Group, P.O. Box 49, H-1525 Budapest, Hungary*

(Dated: June 3, 2025)

* li.song@csrc.ac.cn
† gali.adam@wigner.hun-ren.hu

SUPPLEMENTARY NOTE 1: COMPLEXES OF BORON-VACANCY WITH FIRST NEIGHBORING SUBSTITUTION

Here, we first evaluate the possible V_B structures with innermost substitution. As mentioned above, the single substitutions C_NV_B and O_NV_B have been discussed elsewhere [1–4]. In this discussion, we will consider double and triple substitutions (see Supplementary Figure 1). The respective wavefunctions of the localized states are shown in Supplementary Figure 2. Following the notations in Ref. 5, OOC exhibits a triplet ground state, characterized by two occupied defect levels in the spin-up (spin-majority) channel and one unoccupied level in the spin-down (spin-minority) channel. The unpaired electrons occupy the out-of-plane b_2 and in-plane a_1 orbitals of the carbon atom, with no optical transition occurring between these localized states. It is noteworthy that the presence of oxygen results in an increase in the size of the boron vacancy. Upon optimizing the OCC structure, we observe that the two carbon atoms form a bond with each other, thus eliminating the dangling bonds from the system. Therefore, OCC adopts a singlet ground state, with an energy difference between the occupied b_2 and unoccupied a_2 states of around 3.66 eV. The wavefunction distribution is predominantly from out-of-plane orbitals of the carbon atoms. In contrast, the OOO defect is also a singlet but does not introduce localized levels inside the band gap. Furthermore, OON has a doublet ground state with two defect levels in the gap, specifically in the spin-down channel. The occupied b_2 and empty a_1 defect levels originate from the out-of-plane and in-plane p-orbitals of nitrogen, respectively. This gives rise to a tiny transition dipole moment of around 0.07 Debye between these states, indicating a relatively weak optical transition. Similarly, OCN is also a spin doublet, akin to the previously reported C_NV_B defect [3, 4], where the carbon atom preferentially bonds to a nearby nitrogen atom. The defect levels originate from an out-of-plane orbital of carbon and, notably, there is no optical transition between the intra-defect levels.

Generally, oxygen effectively passivates the dangling bonds of the neighboring atoms without introducing localized states within the gap. In contrast, carbon and nitrogen atoms in CCN and CCC give rise to a greater number of defect levels in the gap. CCN is a doublet with one a_1 unpaired state. Its electronic structure is a combination of those of OCC and OON. Since the optical transition between b_2 and a_1 is weak, the bright emission comes from the optical transition between b_2 and a_2, and we assume that its ZPL energy is quite close to that of OCC. The CCC has a triplet ground state with D_{3h} symmetry, similar to that of V_B^-. The calculated ZPL is at 1.77 eV, and this might decrease once the applied symmetry restriction in the calculations is lifted.

Although these defects are interesting on their own, often introducing a spin-active ground state, we conclude that these complexes of boron vacancy with first-neighbor atom substitution do not produce bright emission at 2 eV, which is often observed in experiments.

SUPPLEMENTARY NOTE 2: CHARGE STABILITY OF CARBON DAP

As shown in Supplementary Figure 3, due to the Coulombic attraction, the total energy of the $C_B^+ - O_NV_B^-$ pair gradually decreases as the distance decreases. We further calculate the formation energy of the $C_B - O_NV_B$ pair in various configurations and find that the Coulombic attraction shifts the charge transition level of the $C_B^+ - O_NV_B^-$ pair compared to the isolated defects [1]. This leads us to conclude that the Coulombic attraction enhances the stability of the $C_B^+ - O_NV_B^-$ pairs over a wider range of Fermi energies.

SUPPLEMENTARY NOTE 3: INTERNAL CONVERSION FROM THE OPTICALLY ACTIVE STATE TO THE METASTABLE DAP STATE

To estimate the non-radiative decay, we use the optically active excited state and the metastable quartet state (Q_s in the main text) as initial and final states to generate the configuration coordinate diagram, assuming that the geometries of the metastable doublet (D_s in the main text) and Q_s are the same. We select C_B5 and C_B7 to extrapolate the distance-dependent electron-phonon coupling. As mentioned in the main text, the fitting function is given by

$$W_{if} = 8.1 \exp(-0.62 R_i), \tag{1}$$

where R_i is the distance between the donor and acceptor in the DAP, as plotted in Supplementary Figure 6. We focus on the > 1.5 nm range, where the electron-phonon coupling is relatively small and yields the order of magnitude of the optical lifetime of the optically active excited state. Since the energy difference between the optically active and metastable DAP states converges to 0.5–0.6 eV in this distance range, we roughly estimate the internal conversion rate based on the configurational coordinates of C_B5 and C_B7. The internal conversion rate $r1$ could reach tens of

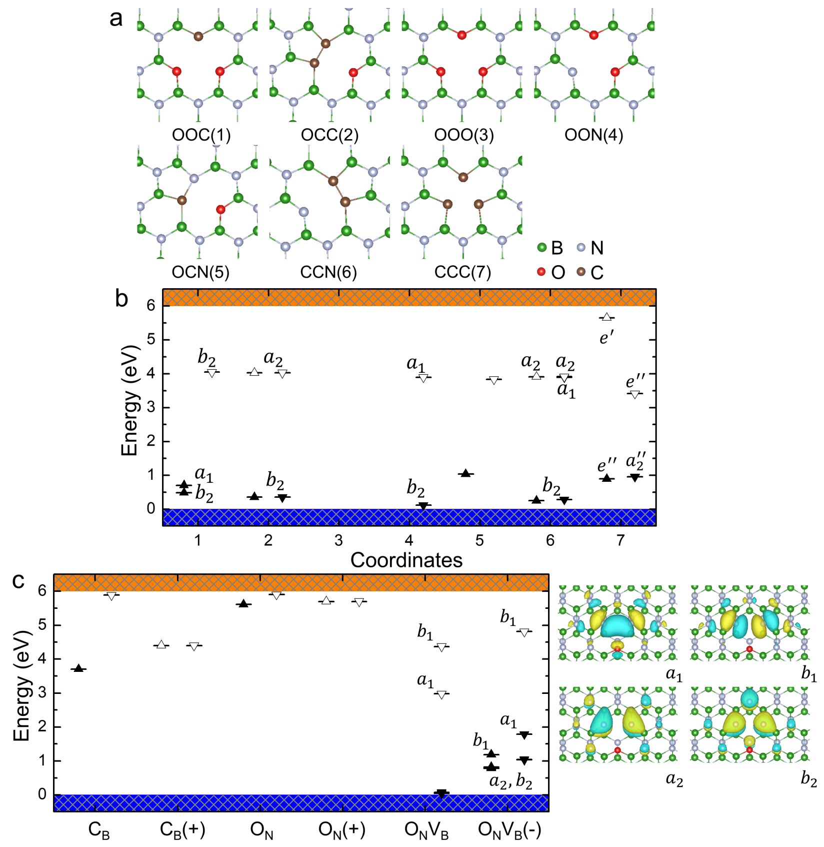

Supplementary Figure 1. The considered defect complexes near the boron vacancy. **a** Schematic view of the optimized structures of carbon- and oxygen-related boron vacancy defects. Only the first-neighbor atoms are replaced by carbon or oxygen, and the symmetry is constrained during optimization. The boron, nitrogen, carbon, and oxygen atoms are depicted as green, grey, brown, and red balls, respectively. **b** The electronic structure of the defects in **a** in the ground state. **c** The electronic structure of carbon and oxygen defects in the ground state. The wavefunctions of the localized states of $O_NV_B{}^-$ are shown.

MHz at ∼1.8 nm, providing a sufficiently long time to mix the spin states of D_s and Q_s at non-zero external magnetic fields. Using the same method, we also estimate the $r3$ process, where the final state is the doublet ground state and the initial state is the metastable state. We find that this $r3$ internal conversion rate is much slower than $r1$.

Supplementary Table I. Calculated non-radiative decay parameters. Energy difference refers to the crossing point between the respective adiabatic potential energy surfaces. ΔQ refers to the change in the coordinates between the initial (i) and final (f) states, whereas the effective phonon modes of the respective states are $\hbar\omega_i$ and $\hbar\omega_f$. Finally, the electron-phonon coupling strength (e-p) is listed.

Configuration	Energy difference (eV)	ΔQ ($\sqrt{\text{amu}}$ Å)	$\hbar\omega_i$ (eV)	$\hbar\omega_f$ (eV)	e-p (eV/$\sqrt{\text{amu}}$Å)
C_B5-r1	0.261	1.056	0.126	0.089	0.284
C_B7-r1	0.287	1.051	0.128	0.091	0.156
C_B5-r2,3	1.793	1.153	0.083	0.081	0.006
C_B7-r2,3	1.686	1.765	0.077	0.075	0.002

SUPPLEMENTARY NOTE 4: OTHER DAP STRUCTURES

Other DAPs also exist in the hBN sample [6]. First, we consider the $C_B - V_B$ pair, since the V_B is a common defect in hBN and has been extensively studied in recent years. Seven kinds of configurations are considered here, as shown in Supplementary Figure 8. In all these configurations, the electron from C_B transfers to V_B making V_B negatively charged. It is easy to identify the state from $C_B{}^+$ (all of them are empty), highlighted in red, and the empty e states

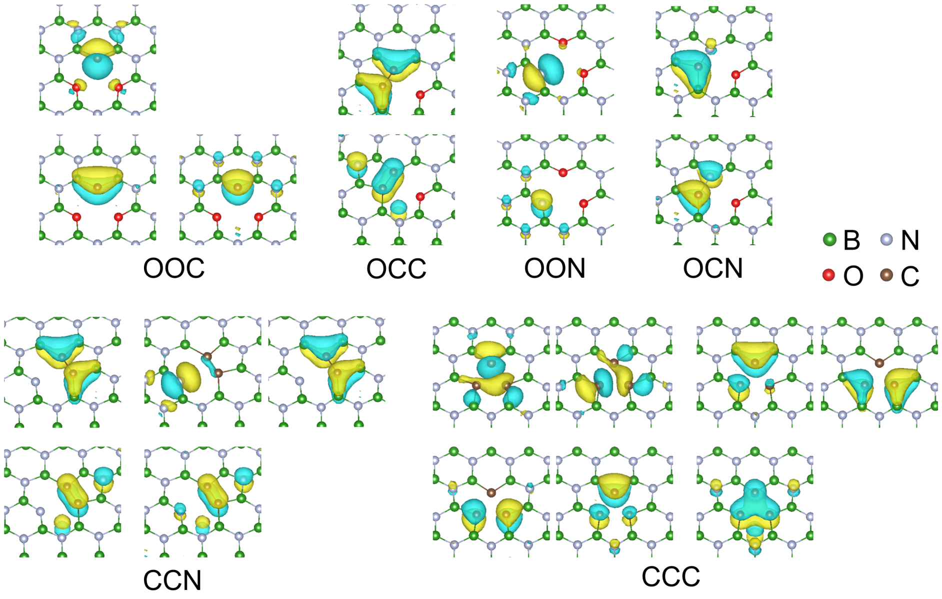

Supplementary Figure 2. Kohn-Sham wavefunctions of complexes of boron vacancy with first-neighbor substitutions. The boron, nitrogen, carbon, and oxygen atoms are depicted as green, grey, brown, and red balls, respectively. The cyan and yellow isosurfaces represent the positive and negative signs of the Kohn-Sham wavefunctions.

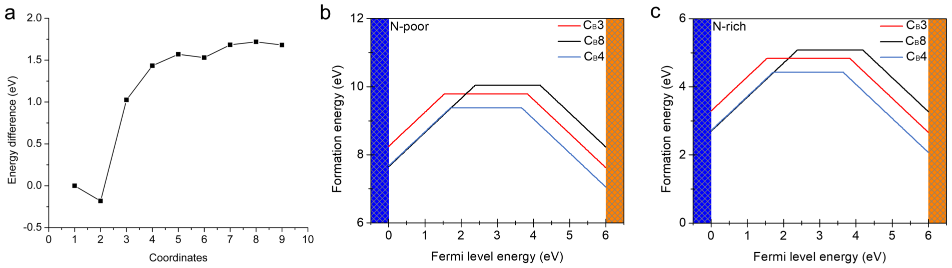

Supplementary Figure 3. **a** The total energy difference of various $C_B - O_N V_B$ pairs calculated using the HSE functional. **b, c** The formation energy of $C_B - O_N V_B$ pairs under N-poor and N-rich conditions. Charge correction is not included here, and we assume that the difference in correction energy for these pairs is small.

from V_B^- (blue). When the two defects are close to each other, the geometry distortion splits the degenerate e states of V_B^-. As the distance increases, the states of C_B^+ shift downward in energy, while the e states shift upward. Using the HSE functional, we observe the crossing of energy levels from the two defects. Similar to $C_B - O_N V_B$, there are two possible spin-conserving excitations here. Nevertheless, the difference is that the local excitation in V_B^- is also a dim transition. Therefore, this DAP system is relatively dark compared to $C_B - O_N V_B$, which might lead to a low ODMR signal. Moreover, it is important to note that the ground state of this DAP is $S = 1$, whereas it is $S = 1/2$ in $C_B - O_N V_B$, so the optical loop discussed in Figure 4 of the main text would be different.

Another example is $C_B - C_N V_B$, which is similar to $C_B - O_N V_B$, and also has a ground state of $S = 1/2$. Previously, we reported the reconstruction of the $C_N V_B$ defect, where the carbon atom bonds with a nearby nitrogen atom to form a Stone-Wales-like geometry [3]. However, we find that the presence of C_B can influence the geometry of $C_N V_B$, as shown in Supplementary Figure 9. Unlike the oxygen atom, the carbon atom still has one dangling bond, so there are more localized states in the gap. It is still possible to identify the empty state from C_B since there is no energy splitting between the spin-up and spin-down channels. Using the HSE functional, we observe that the empty state from C_B shifts downward, while the empty state from $C_N V_B$ shifts upward. Similar to $C_B - O_N V_B$, this kind of level crossing indicates that the charge transfer could influence the optical excitation, and the model proposed in Figure 4 of the main text still holds.

Other DAPs, for example $C_B - C_N$, have also been investigated recently [7]. The difference in that case is that there is just one localized state from each defect, and the total spin is $S = 0$. Therefore, the optical transition is only associated with the charge transfer process.

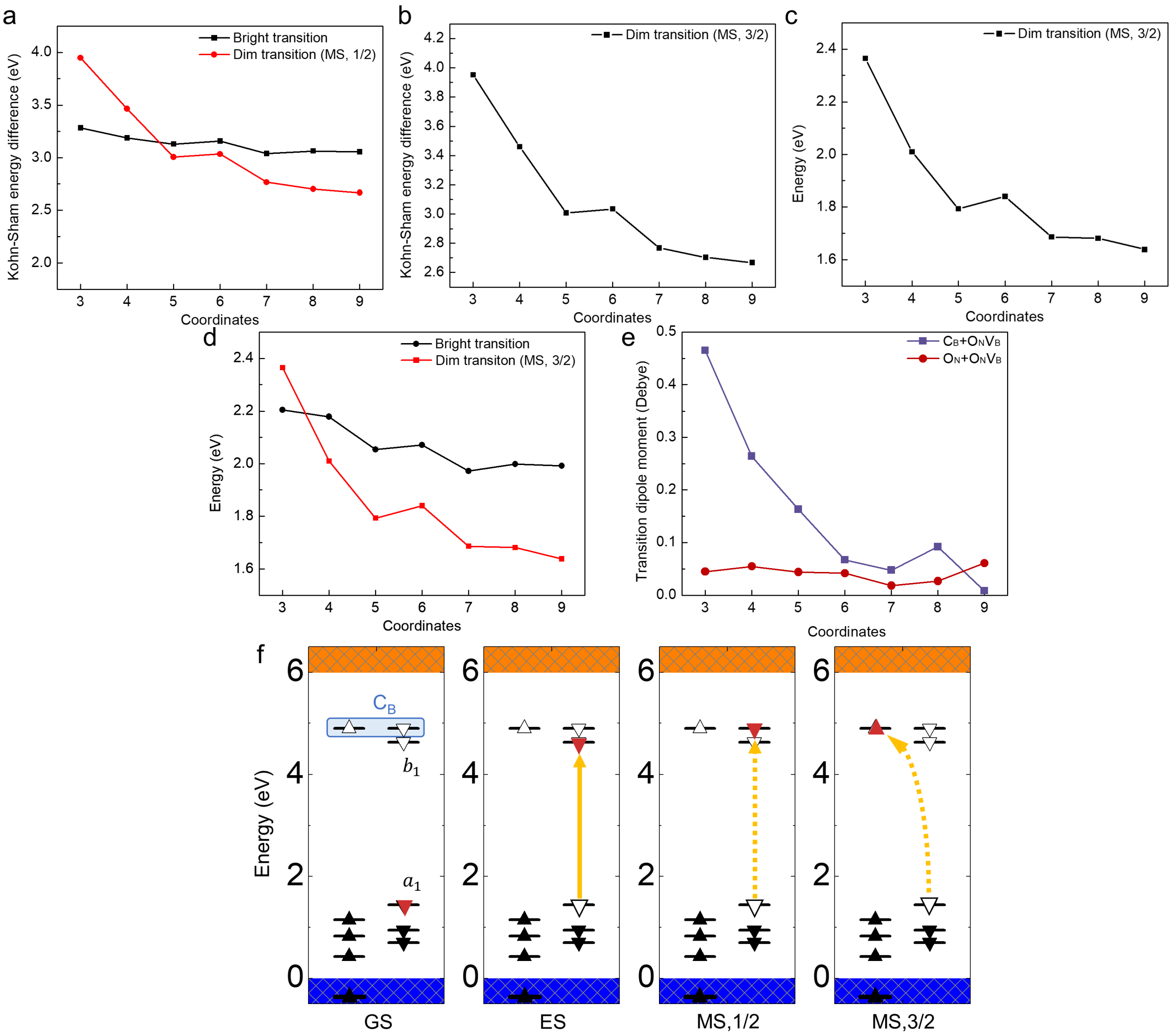

Supplementary Figure 4. The energy difference between bright and dim transitions in different configurations. **a, b** The Kohn-Sham energy difference for the bright optical transition within $O_NV_B^-$ and the dim optical transition from $O_NV_B^-$ to C_B^+. **c, d** The calculated transition energies for the bright optical transition within $O_NV_B^-$ and the dim optical transition from $O_NV_B^-$ to C_B^+. **e** The transition dipole moment of the dim transition. **f** The electron occupation of C_B4 in the ground state (GS), excited state (ES), and metastable state (MS). Filled and unfilled triangles indicate empty and occupied defect levels in the gap, and the triangle direction denotes spin majority or minority. The red triangle indicates the a_1 orbital of O_NV_B considered for excitation. The solid and dashed orange lines represent the bright and dim excitation paths.

SUPPLEMENTARY NOTE 5: OXYGEN IN LARGER VACANCIES

Oxygen impurities may exist in multi-vacancy structures. Given that the O_B defect has a higher formation energy, we simply modeled three larger vacancies containing the O_N defect, as shown in Supplementary Figure 10. It is evident that the neighboring boron atoms bond with each other, while the nitrogen atoms do not. In defect 1, the paired boron dimer introduces an out-of-plane orbital in the gap, while the other orbitals originate from the dangling bond on the nitrogen atom. In defect 2, the localized states solely arise from two sets of paired boron dimers. In defect 3, in addition to the paired boron dimer, localized states arise from the unpaired single boron atom, while the remaining states come from two unsaturated nitrogen atoms, similar to those of the O_NV_B defect. Consequently, the possible optical excitation is analogous to that of the O_NV_B defect, and the calculated ZPL is around 2.5 eV.

We propose that multi-vacancy systems without oxygen or carbon doping warrant further study. Nevertheless, a comprehensive investigation would require examining tens of possible configurations regarding the size of the multi-vacancy, which is beyond the current research scope.

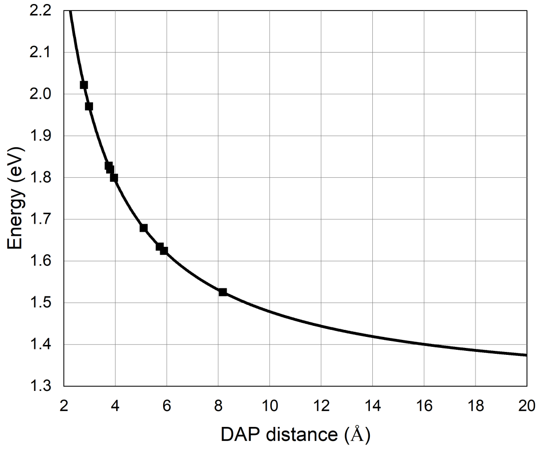

Supplementary Figure 5. Distance-dependent charge transfer dim emission energy as a function of R_i, based on the DAP model for $C_B - O_N V_B$. The squares indicate the geometries calculated in the main text.

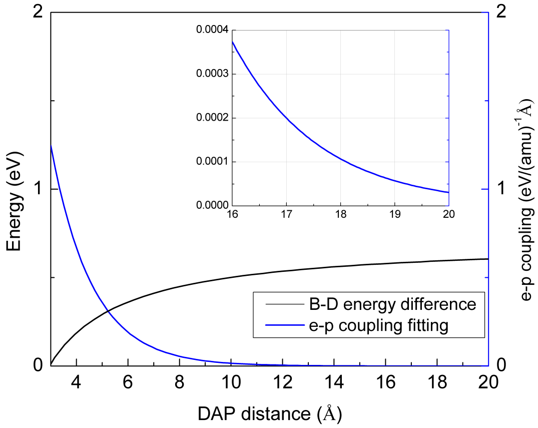

Supplementary Figure 6. Distance-dependent energy difference between the intrinsically bright and dim transitions (black) and the electron-phonon coupling strength (blue).

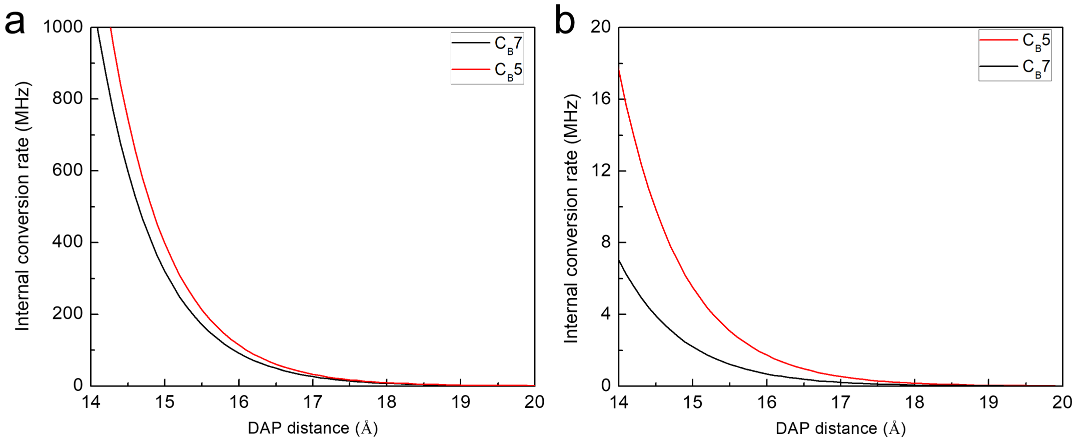

Supplementary Figure 7. Distance-dependent internal conversion rate based on configurational coordinates from DFT calculations. **a** $r1$ rate, **b** $r3$ rate.

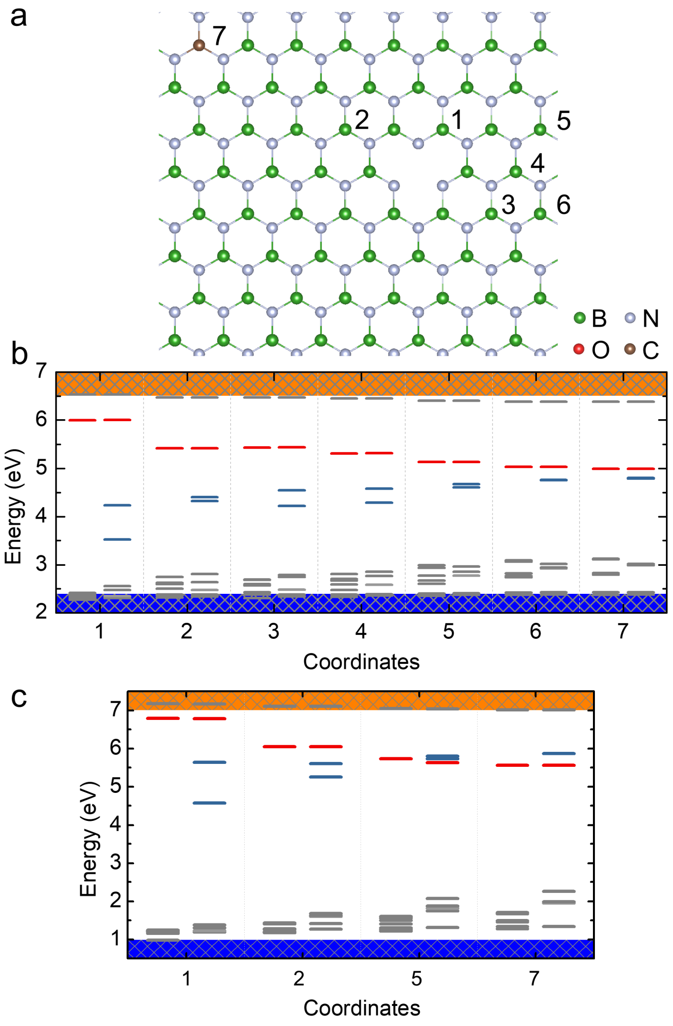

Supplementary Figure 8. The $C_B - V_B$ DAP system and its electronic structure in the ground state. **a** The configurations we considered. **b** The energy levels in the ground state using the PBE functional. **c** The energy levels in the ground state using the HSE functional. The red line denotes the empty states from ${C_B}^+$, and the blue line denotes the e states from ${V_B}^-$.

SUPPLEMENTARY NOTE 6: CONVERGENCE TEST

We tested the cutoff energy using the PBE functional in a primitive hBN cell, as shown in Supplementary Figure 11. The energy difference between 450 eV and 600 eV is within 5 meV, so we set it to 450 eV to accelerate the computation of excited states. In addition, the mixing parameter α influences the band gap. We also calculated the ZPL of negatively charged O_NV_B for various mixing fractions. The band gap increases from 5.9 to 6.4 eV, but the ZPL increases by only $\sim$0.1 eV, so the mixing parameter does not significantly influence the optical properties of the defect at this level.

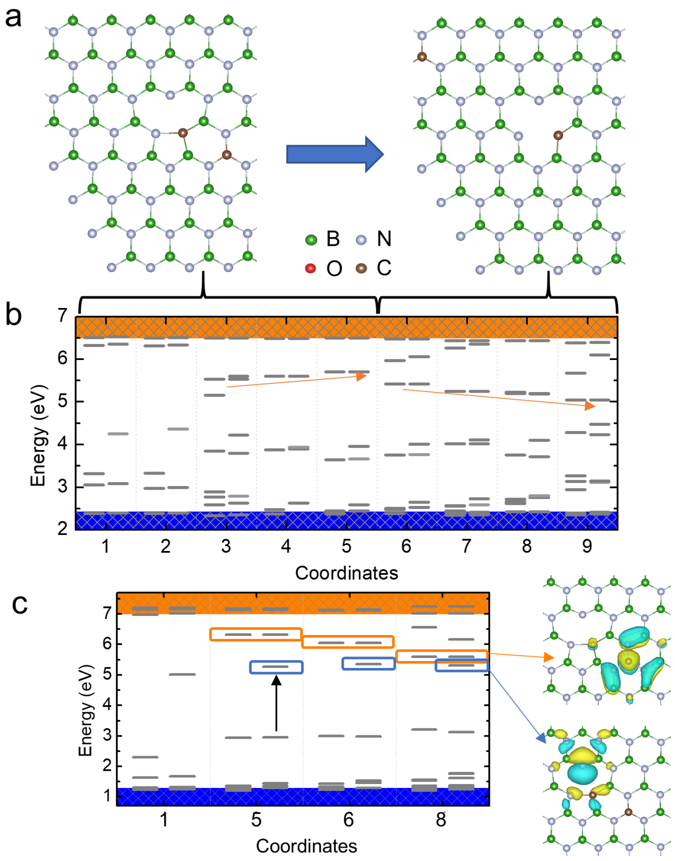

Supplementary Figure 9. The $C_B - C_N V_B$ DAP system and its electronic structure in the ground state. **a** The configurations we considered. **b** The energy levels in the ground state using the PBE functional. **c** The energy levels in the ground state using the HSE functional.

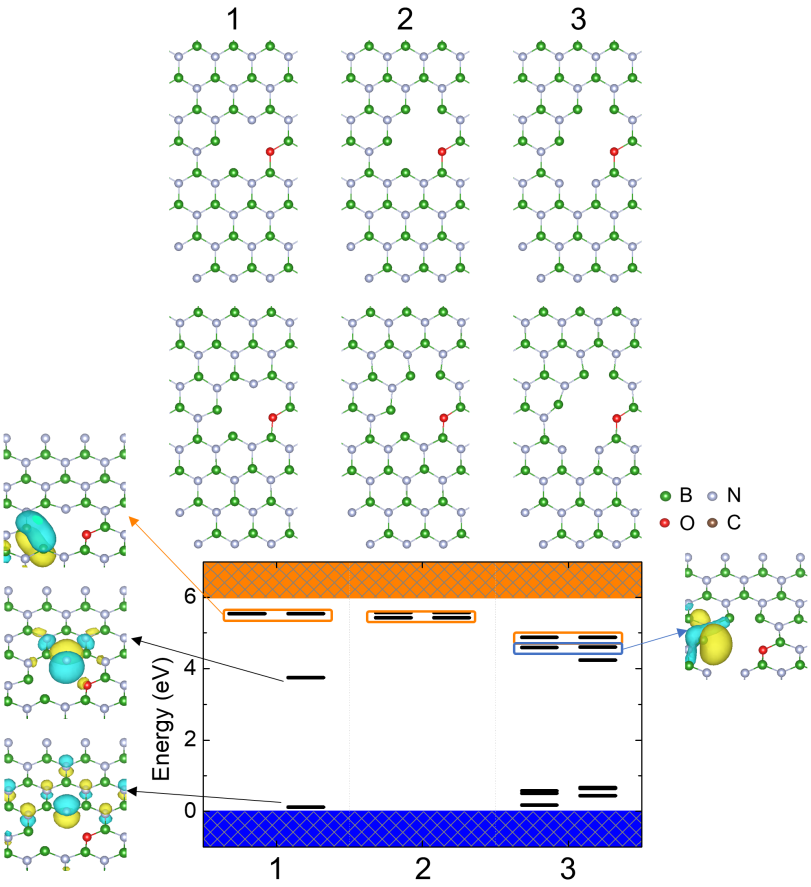

Supplementary Figure 10. The multi-vacancy systems with the O_N defect. The states labeled in orange originate from the neighboring boron dimer. The states labeled in blue are from the single boron dangling bond. The rest originate from the nitrogen dangling bond.

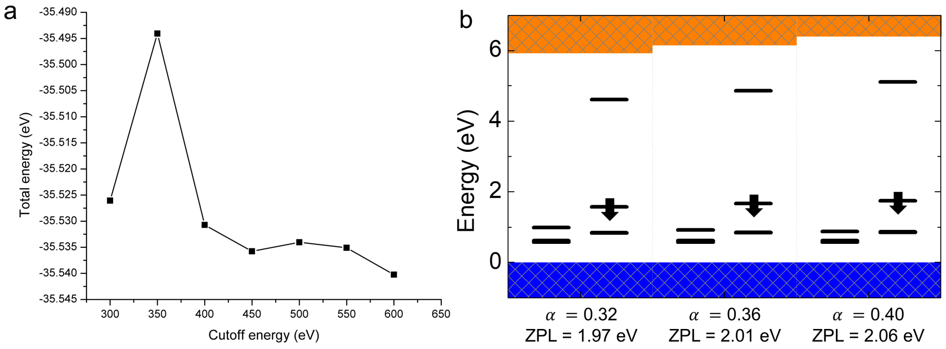

Supplementary Figure 11. **a** Test of the cutoff energy in the hBN primitive cell using the PBE functional. **b** The influence of the mixing parameter on the ZPL of $O_NV_B^-$.

SUPPLEMENTARY NOTE 7: REFERENCE

[1] L. Weston, D. Wickramaratne, M. Mackoit, A. Alkauskas, and C. G. Van de Walle, Phys. Rev. B **97**, 214104 (2018).
[2] S. Li and A. Gali, J. Phys. Chem. Lett. **13**, 9544 (2022).
[3] S. Li and A. Gali, Front. Quantum Sci. Technol. **1**, 1007756 (2022).
[4] M. A. Ortigoza and S. Stolbov, Phys. Rev. B **105**, 165306 (2022).
[5] S. X. Li, T. Ichihara, H. Park, G. He, D. Kozawa, Y. Wen, V. B. Koman, Y. Zeng, M. Kuehne, Z. Yuan, *et al.*, Commun. Mater. **4**, 19 (2023).
[6] Q. Tan, J.-M. Lai, X.-L. Liu, D. Guo, Y. Xue, X. Dou, B.-Q. Sun, H.-X. Deng, P.-H. Tan, I. Aharonovich, *et al.*, Nano Letters **22**, 1331 (2022).
[7] P. Auburger and A. Gali, Phys. Rev. B **104**, 075410 (2021).
